# Identification of volatiles from *Pinus silvestris* attractive for *Monochamus galloprovincialis* using a SPME-GC/MS platform

**DOI:** 10.1007/s11356-012-0792-5

**Published:** 2012-02-10

**Authors:** Rafal Szmigielski, Marek Cieslak, Krzysztof J. Rudziński, Barbara Maciejewska

**Affiliations:** 1 Laboratory of Environmental Chemistry, Institute of Physical Chemistry, Polish Academy of Sciences, 44/52 Kasprzaka St, 01-224 Warszawa, Poland; 2 CHEMIPAN R&D Laboratories, Institute of Physical and Organic Chemistry Polish Academy of Sciences, 44/52 Kasprzaka St, 01-224 Warszawa, Poland

**Keywords:** Solid-phase micro-extraction (SPME), Gas chromatography/mass spectrometry (GC/MS), Terpenoids, *Pinus sylvestris*, *Monochamus galloprovincialis*, Insect, Chemical signalling

## Abstract

**Introduction:**

A myriad of volatile organic compounds (VOCs) released by terrestrial vegetation plays an important role in environmental sciences. A thorough chemical identification of these species at the molecular level is essential in various fields, ranging from atmospheric chemistry to ecology of forest ecosystems. In particular, the recognition of VOCs profiles in a context of plant–insect communication is a key issue for the development of forest protection tools.

**Purpose:**

This work was aimed at the development of a simple, robust and reliable method for the identification of volatiles emitted from plant materials, which can attract or deter pest insects. Specifically, volatiles emitted from the bark of *Pinus sylvestris* were studied, which might attract the black pine sawyer beetle *Monochamus galloprovincialis*—a serious pest of the tree and a vector of a parasitic nematode *Bursaphelenchus xylophius.*

**Method:**

The volatiles from bark samples were collected using a solid-phase micro-extraction technique, and subsequently analysed by gas-chromatography/mass-spectrometry (GC/MS). The characterisation of the volatile fraction was based on the comparison with data in mass spectral libraries, and in most cases, with the available authentic standards. The identified compounds were screened against the available entomological data to select insect attractors.

**Results:**

The identified components included terpenes (α-pinene, ∆-3-carene, and para-cymenene), oxygenated terpenes (α-terpineol and verbenone), sesquiterpenes (α-longipinene, longifolene, E-β-farnesene, γ-cadinene and pentadecane), and diterpenes (manoyl oxide and (+)-pimaral). Of these, longifolene and (+)-pimaral are of particular interest as plausible attractors for the *M. galloprovincialis* beetle that might find application in the construction of insect bait traps.

## Introduction

The chemical communication between animals and plants has received a lot of interest by the scientific community for years (Dicke et al. 1990; Felton and Tumlinson 2008; Zheng and Dicke 2008). These interactions might benefit either the emitting or receiving species; however, our knowledge in this field is yet considerably constrained. Limitations arise from several reasons, including complexity of the chemical composition of “a molecular cocktail released”, a poorly recognised chemical and physiological nature and an extremely low concentration range of numerous signalling molecules. As far as the latter is concerned, the laboratory studies showed that just a few molecules were enough to elicit a behavioural response in a receiving species. Field experiments with bombykol—a sex pheromone of the silkworm moth *Bombyx mori* have revealed that *ca*. 200 molecules per 1 mL of air are enough to trigger a response in a downwind receptive male (Herrmann 2010). Low detection limits and, in most cases, the lack of reference materials make the analysis of signalling molecules a real challenge. However, micro-extraction techniques, such as solid-phase micro-extraction (SPME), combined with mass spectrometry offer a reasonable solution in this matter (Belliardo et al. 2006; Yassaa and Williams 2007).

**Fig. 1:**
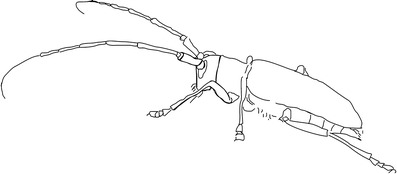
Black sawyer beetle *M. galloprovincialis*—a ubiquitous pest of the Scots pine (*P. sylvestris*) forests

The objective of our work was to develop a SPME-gas chromatography/mass-spectrometry (GC/MS) method for the identification of the volatile organic fraction from the bark of Scots pine (*Pinus sylvestris*) and thereby to select the most promising molecular candidates attractive for *Monochamus galloprovincialis*—the ubiquitous pest of European and Asian forests (Fig. 1). The scope of this study falls into the policy guidelines of the Food and Agriculture Organisation of the United Nations on the development of forest pest management strategies through a design of novel dispensers and trap baits for pest control (FAO 1995).

*P. sylvestris* is an evergreen coniferous tree native to vast areas of Europe and Asia, ranging from Scotland, Ireland and Portugal in the west, to Eastern Siberia in the east, the Caucasus Mountains in the south, and the Arctic Circle in Northern Scandinavia (Farjon 2005). The emission profile of volatile organic compounds (VOCs) from *P. sylvestris* was mostly measured in natural conifer forests in the context of atmospheric sciences (Isidorov et al. 2003; Kesselmeier and Staudt 1999; Tarvainen et al. 2005). The field measurements were carried out either above or under forest canopies, so they delivered overall emission fluxes from several tree species. The main emitted compounds were monoterpenes, such as ∆-3-carene, α-pinene and β-pinene which constituted 60–85% of the total observed monoterpene emission rates. In addition, oxygenated isoprenoids, such as 2-methyl-3-buten-2-ol and sesquiterpenes, mainly β-caryophyllene were observed, depending on the season. The emission measurements were carried out using a dynamic flow through technique with samples collected on adsorbent tubes and analysed using thermodesorption followed by GC/MS (Tarvainen et al. 2005). Direct emission of volatiles from *P. sylvestris* were measured at the seedling (Hao et al. 2009), shoot (Ruuskanen et al. 2005; Back et al. 2005; Hao et al. 2009), branch (Komenda et al. 2001; Yassaa and Williams 2007) and needle (Mateus et al. 2010) levels. The latter authors analysed the emissions using headspace solid-phase microextraction and steam distillation extraction as well as one- and two-dimensional gas chromatography with time-of-flight mass spectrometric detection (Mateus et al. 2010). Their supplementary material contains an extensive list of identified components including enantiomeric forms of compounds. Bark of *P. sylvestris* has been used for biomonitoring of pollutants (Swieboda and Kalemba 1979; Pöykiö et al. 2005), but to our best knowledge, it has not been studied separately for emission of VOCs with the exception of stem bark of 2-year-old seedlings (Heijari et al. 2011).

The relevant information regarding the volatile fraction from *P. sylvestris* stems from the comprehensive studies on the composition of the essential oils prepared from needles or roots of the plant (Maciag et al. 2007). Based on head-space (HP) GC/MS, it was found that the composition of the volatile fraction considerably varied depending on the source of the oil. In the essential oils from the Pine roots, about 20 volatile compounds were detected with α-pinene (36%) and ∆-3-carene (36%) as dominant monoterpene constituents. Other relatively abundant constituents included β-pinene, longifolene, sabinene, terpinolene, limonene, β-phellandrene, which together amounted to 20% (Napierała-Filipiak et al. 2002). Interestingly, a far richer composition of the volatile fractions was observed for oils from the *P. sylvestris* needles. In this case, more than 70 individual compounds were characterised, including monoterpenes such as α-pinene, ∆-3-carene, camphene and myrcene as well as sesquiterpenes such as ∆-cadinene (Maciag et al. 2007). Different compositions of the aroma flavour of the essential oils could result from different preparation methods during which a partial loss of volatiles could happen.

The study on VOCs emitted from the *P. sylvestris* timber showed that terpenes were clearly the main compound group for air-dried wood samples, whereas aldehydes, carboxylic acids and their esters dominated for heat-treated wood samples (Manninen et al. 2002). Fourteen individual compounds out of 41 identified ones were found in both types of wood samples, indicating considerable changes in the VOC emission profile during the wood treatment processes.

Interestingly, some of the emitted VOCs, or combinations thereof can attract insect herbivores to choose *P. sylvestris* as a host tree for feeding and reproduction. More specifically, it was observed that *P. sylvestris* is a host tree for a serious secondary pest—the black sawyer beetle *M. galloprovincialis* (*Coleoptera*: *Cerambycidae*; Pajares et al. 2004, 2010). The pest attacks and colonises the weakened, dying or recently cut trees and—when aggregated in large assemblies—attacks the healthy ones. Not only feed the adult insects on *Pinus* tree needles but also bore holes in the bark to oviposition. The larvae develop under the bark and subsequently enter the sapwood and heartwood, constructing systems of complex galleries that profoundly weaken the trees. In addition, the pine sawyer is a vector of the pinewood nematode *Bursaphelenchus xylophius*, which causes the pine wilt disease (Naves et al. 2008). Altogether, *M. galloprovincialis* poses a serious threat to pine forests. For this reason only, it is very important to understand the mechanisms by which *P. sylvestris* attracts this dangerous pest.

To the best of our knowledge, this problem is vaguely recognised and deserves scientific attention. In spite of the broad collection of emission data resulting from the aforementioned researches, the understanding of chemical interaction between *P. sylvestris* and *M. galloprovincialis* is limited. We know that several emitted compounds worked as components of kairomonal lures tested in pest management (Ibeas et al. 2007; Pajares et al. 2004, 2010; Francardi et al. 2009). Perception of *P. sylvestris* odours (whole plant bouquet) was studied in *M. galloprovincialis* (Weißbecker et al. 2006), and in other herbivore insects (Byers et al. 1985; Sakai and Yamasaki 1990). The presented state of knowledge justified the objectives of this work, in which mass spectrometry was a first choice method for the analysis of the bark volatiles collected by the SPME sampling technique.

## Experimental

### Sample preparation and experimental setup

**Fig. 2:**
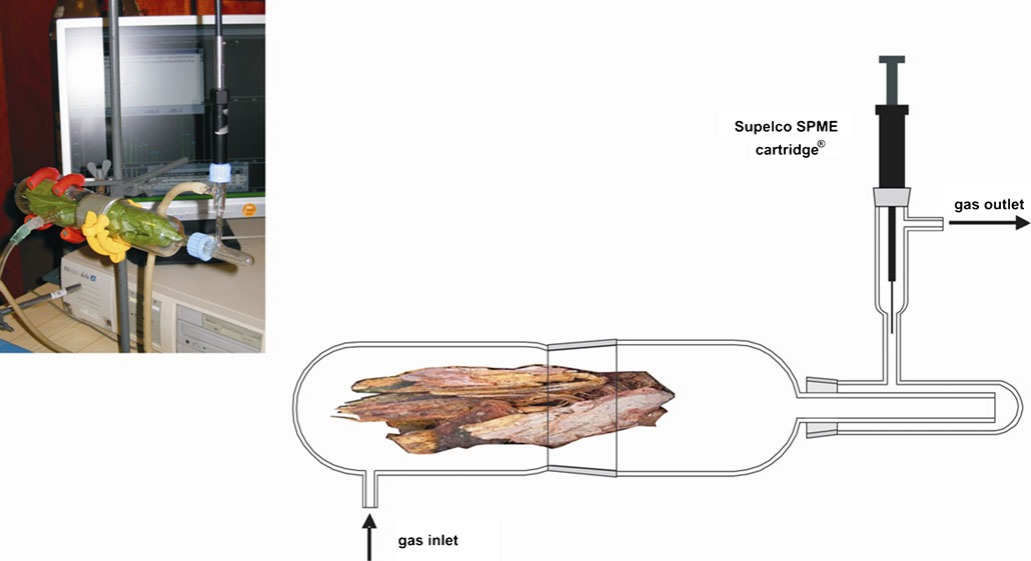
Schematic representation of a sampling flow apparatus equipped with a dynamic solid-phase micro-extraction (SPME) needle cartridge with a 100 μm polidimethylsiloxane fibre coating used in the study; the inset shows the photograph of the experimental set up

Experimental setup used included a home-made sampling flow apparatus with an SPME needle cartridge and two gas chromatographs: an HP6890 model with a HP5973 single quadrupole electron ionisation mass selective detector (GC/MS) and an HP6980 model with a flame ionisation detector (GC/FID), all from Agilent Technologies. The sampling flow apparatus consisted of a horizontal flow tube, through which an extracting gas was pumped at a controlled velocity (Fig. 2). A sample for extraction was placed in the inlet section of the tube. The outlet section of the tube contained a perpendicular port through which an SPME needle cartridge was inserted. After extraction, the SPME needle cartridge was placed directly in the split/splitless injector of the GC/MS or GC/FID apparatus for thermal desorption and analysis, the details thereof given in the following sections.

Branches of young *P. sylvestris* of diameters ranging from 2 to 7 cm were collected in a conifer forest near Warsaw, Poland, wrapped in aluminium foil and transported to the laboratory within 1 day. Bark samples approximately rectangular and 2 × 10 cm in size were taken manually from the branches and immediately placed in the extracting apparatus. Extraction of volatiles from each sample was carried out for 2 h at room temperature, with clean air flowing at a rate of 10 mL/min. Altogether, 10 bark samples were processed and analysed.

### GC/MS analysis

Immediately after sampling, the SPME needle was introduced into the split/splitless injector of the GC/MS instrument fitted with the HP-5MS fused silica capillary column (30 m × 0.25 mm i.d. × 0.25 μm; crosslinked 5% Phenyl-Methylpolysiloxane). Desorption was achieved in splitless mode at 250°C for 30 s. These settings were sufficient for a quantitative desorption of all the analytes studied. This was checked by subjecting the SPME fibre to a second run, which always showed no carry-over peaks. The MS system was operated in the scan mode (30–550 u) under the following conditions: ionisation potential, 70 eV; source temperature, 230°C; and MS quadrupole temperature, 150°C. The carrier gas was helium (1.0 mL/min, AirLiquide, Poland). The temperature programme was adopted from our previous study (Szmigielski et al. 2007) and slightly modified as follows: the isothermal hold at 50°C for 5 min, the temperature ramp of 10°C/min up to 270°C, the isothermal hold at 270°C for 5 min. Compounds were identified based on the comparison with data in NIST/EPA/NIH Mass Spectral Library included with the MSD ChemStation software of the 7890A GC System and Wiley-VCH Verlag Mass Spectral and GC Data library (Pfleger et al. 2007). In addition, the identification of the unknown compounds was supported using the comparison of their mass spectral data and chromatographic retention times against the commercially available synthetic standards (Sigma-Aldrich, Across Organics, Alfa-Aesar, BOC Sciences). In order to minimise the risk of misinterpretation, acquired mass spectra were additionally compared against other open-access electronic mass spectra libraries, including: (1) Pherobase library (http://www.pherobase.com), (2) NIST Chemistry WebBook (http://webbook.nist.gov), and (3) SDBS Spectral Database for Organic Compounds (http://riodb01.ibase.aist.go.jp/sdbs/cgi-bin/direct_frame_top.cgi).

### GC-FID analysis

In order to assess the contribution of individual components, a few collections of volatiles were additionally analysed by the GC/FID instrument fitted with a split/splitless injector and the HP-35 fused silica capillary column (30 m × 0.25 mm i.d. × 0.25 μm; crosslinked 35% Phenyl-Methylpolysiloxane). The carrier gas was nitrogen (1.0 mL/min, Multax, Poland). The injector was operated in the split mode with the split ratio of 1.0, and heated to the temperature of 250°C. The FID parameters were as follows: makeup flow, 28 mL/min; air flow, 350 mL/min; and hydrogen flow, 35 mL/min. The oven temperature programme was the same as in the GC/MS analyses described in the preceding paragraph.

## Results and discussion

**Fig. 3:**
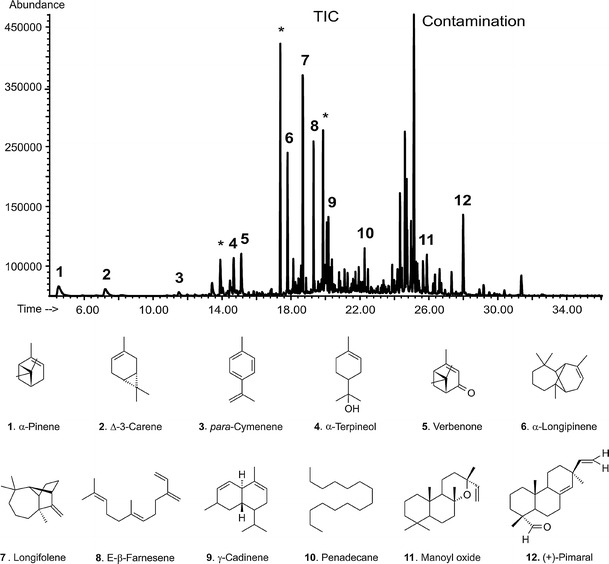
A representative SPME-GC/MS profile of the volatiles from the *P. sylvestris* bark, and chemical structures of the identified compounds 1–12

Figure 3 shows a representative GC/MS total ion current chromatogram (TIC) obtained for the solid-phase micro-extracted volatile fraction from the *P. sylvestris* bark. Comparable distribution profiles of the volatile fraction have been observed in all samples, although slight variations in the relative intensities of chromatographic peaks corresponding to the same constituents in different samples were observed. These could result from different content of organic compounds in the bark, depending on its age. As indicated in Fig. 3, a volatile bouquet from the *P. sylvestris* bark represents a complex mixture of low-molecular mass compounds. A first glimpse on the chromatogram allows selecting the representative retention-time zones (RTZ).

**Fig. 4:**
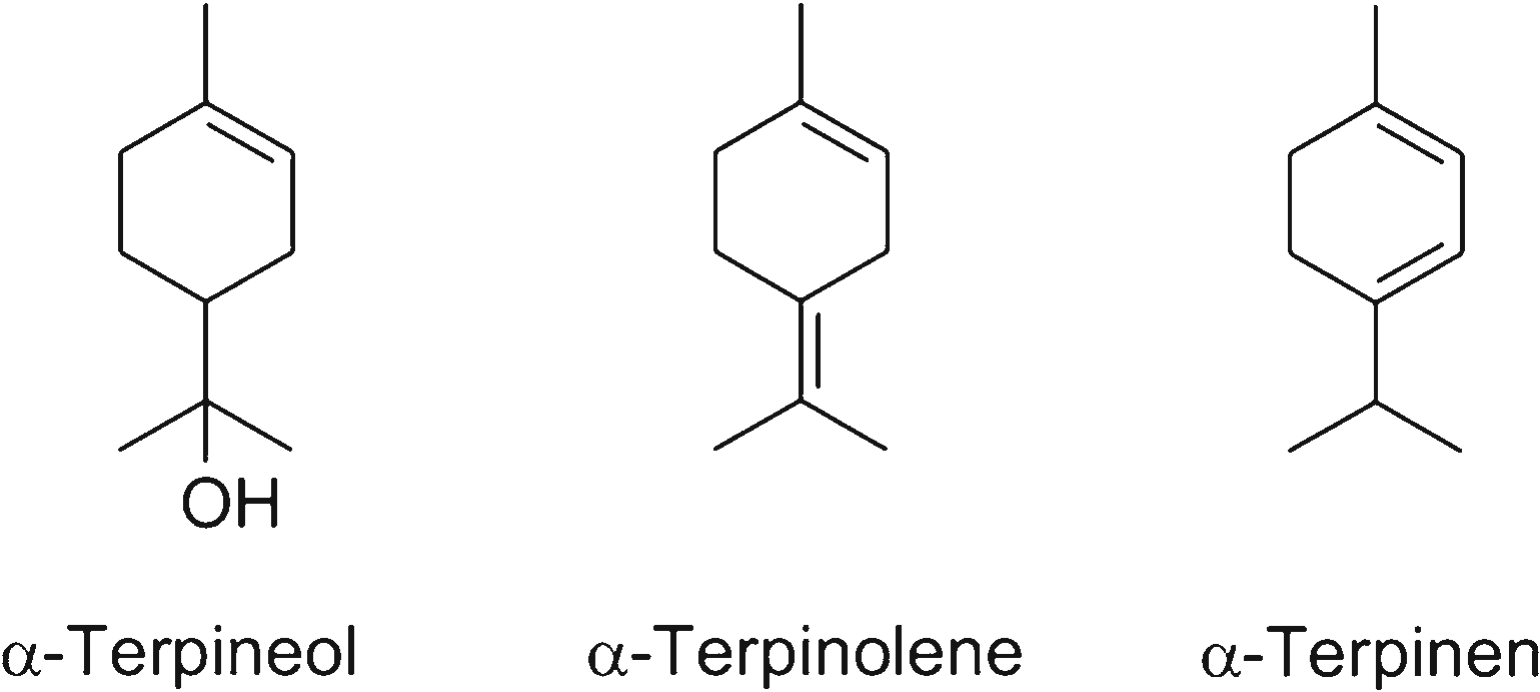
Structures of important volatiles from Scots pines and other conifer trees

The first RTZ covers the region up to 15 min and contains five different and well baseline-resolved compounds. Compounds 1, 2 and 3 were identified as α-pinene, ∆-3-carene and para-cymenene, respectively and represent terpenes—an important group of plant volatiles. It should be noted that the elution of ∆-3-carene is followed directly by the elution of DL-limonene—another important terpene, which appears in the chromatogram as a hardly visible peak at the retention time of 8.2 min. Previously, these compounds were reported as important and ubiquities volatiles from *P. sylvestris*, both in the context of atmospheric research (Isidorov et al. 2003; Kesselmeier and Staudt 1999; Tarvainen et al. 2005), the essential oils (Maciag et al. 2007) and the *Pinus* timber seasoning (Manninen et al. 2002). Based on the GC-FID results, the relative contribution of these monoterpenes to the volatile fraction from the *Pinus* bark was estimated to be as high as 24%. This assessment is in good agreement with the emission profile from Scots pine (*P. sylvestris*) shoots caused by the large pine weevil *Hylobius abietis* insect reported by Heijari et al. (2011). Compounds 4 and 5 were identified as α-terpineol and verbenone, respectively, and both fall into a group of the so-called oxygenated terpenes. As far as the identification of α-terpineol 4 is concerned, the precaution should be taken when using the Wiley mass spectral library. In the EI mass spectrum of 4 (MW 154), the molecular ion is not visible while the first important fragment ion emerges at *m/z* 136 that corresponds to the odd electron [M−H_2_O]^+▪^ ion. This is basically a primary source of errors in the library-based identification of the structurally related volatiles, i.e., α-terpinolene and α-terpinen (Fig. 4) that are just dehydratated derivatives of α-terpineol 4 (tertiary alcohol) which appear at considerable concentrations in the environmental samples.

**Fig. 5:**
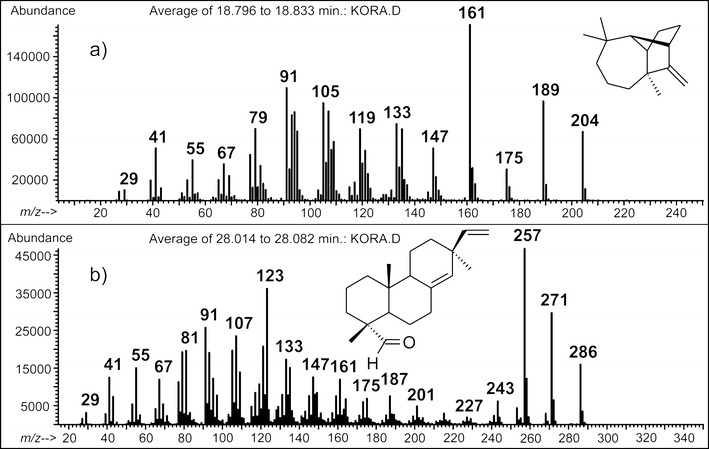
EI mass spectra of the plausible attractant compounds for black sawyer beetle *M. galloprovincialis*: **a** longifolene and **b** (+)-pimaral

**Scheme 1:**
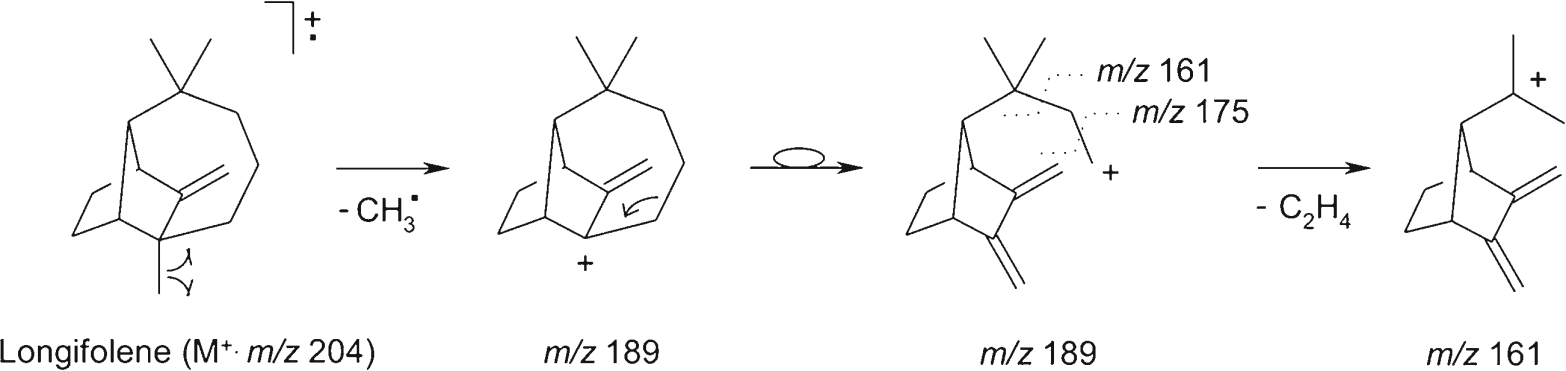
A plausible fragmentation channel leading to the diagnostic ions in the EI mass spectrum of longifolene **7**

The second retention time zone (Fig. 3), ranging from 15 to 22 min, is characteristic of the elution of a group of five compounds with the identical molecular mass (MW 204). The comparative analysis of their mass spectral and chromatographic behaviour with that of the synthetic standards firmly allowed to infer their molecular identity: α-longipinene 6, longifolene 7, E-β-farnesene 8, γ-cadinene 9 and pentadecane 10. Additionally, Kovats retention indices (KRI) calculated for these species are in line with data published in the literature (NIST Chemistry WebBook, http://webbook.nist.gov; and the Pherobase, http://www.pherobase.com). For instance, the KRI value determined for the compound eluting at 19.44 min using our HP-5MS column and under conditions expressed in the experimental part (Section 2.1) was 1,432 whereas that published in the NIST Chemistry WebBook—1,439 based on the measurements with HP-5MS column under fairly equivalent GC conditions (Gkinis et al. 2003). The KRI indices were calculated from injection of C11 to C19 *n*-alkanes on an HP-5MS stationary phase. The group of sesquiterpenes identified accounts for *ca*. 48% of the volatile fraction from the bark of *P. sylvestris*. Our observation lies in a stark contrast to the findings of Heijari et al. who noticed a considerable contibution from other sesquiterpenes, namely β-bourbonene and β-caryophellene (Heijari et al. 2011). This inconsistence can be explained by different maturation levels of *P. sylvestris* used in both studies. Longifolene **7** along with γ-cadinene 9 dominate in the composition of the essential oils from the needles of *P. sylvestris* (Maciag et al. 2007). The EI mass spectrum of longifolene **7** displays an interesting set of fragment ions, with the *m/z* 161 ion as the most abundant one (Fig. 5a). The plausible fragmentation reaction leading to this ionic product is initiated by a methyl radical loss, likely at a C-7 position (Scheme 1). This step affords the formation of the first relevant fragment ion (*m/z* 189). Following the nitrogen rule, it is wise to assume that the latter ion must undergo a skeletal rearrangement prior to a further decomposition reaction. The positive charge undergoes neutralisation and the seven-membered ring opens easily. In the next step, the combined losses of the two methylene moieties have to take place, leading to the formation of a thermodynamically stable (tertiary) carbocation (*m/z* 161)—the most dominating ion in the longifolene **7** mass spectrum. Its structure readily explains the presence of other ions at the lower *m/z* region of mass spectrum (i.e., *m/z* 147, *m/z* 133 and *m/z* 119) via the mechanism typical of aliphatic and acyclic hydrocarbons (McLafferty and Turecek 1993). The proposed fragmentation pattern of longifolene **7** aids the correct understanding the EI mass spectra for other Pine wood sesquiterpenes with a tricyclic skeleton. E-β-farnesene 8 and pentadecane 10 feature the characteristic fragmentation reactions, typical of linear and saturated hydrocarbons (McLafferty and Turecek 1993). As proved by SPME-GC/MS analysis, γ-cadinene 9 is a significant constituent of the essential oils from needles of *P. sylvestris* (Kula et al. 1996; Maciag et al. 2007). Interestingly, this sesquiterpnene is emitted in the significant amounts by Pine twigs (Mumm and Hilker 2005).

**Scheme 2:**
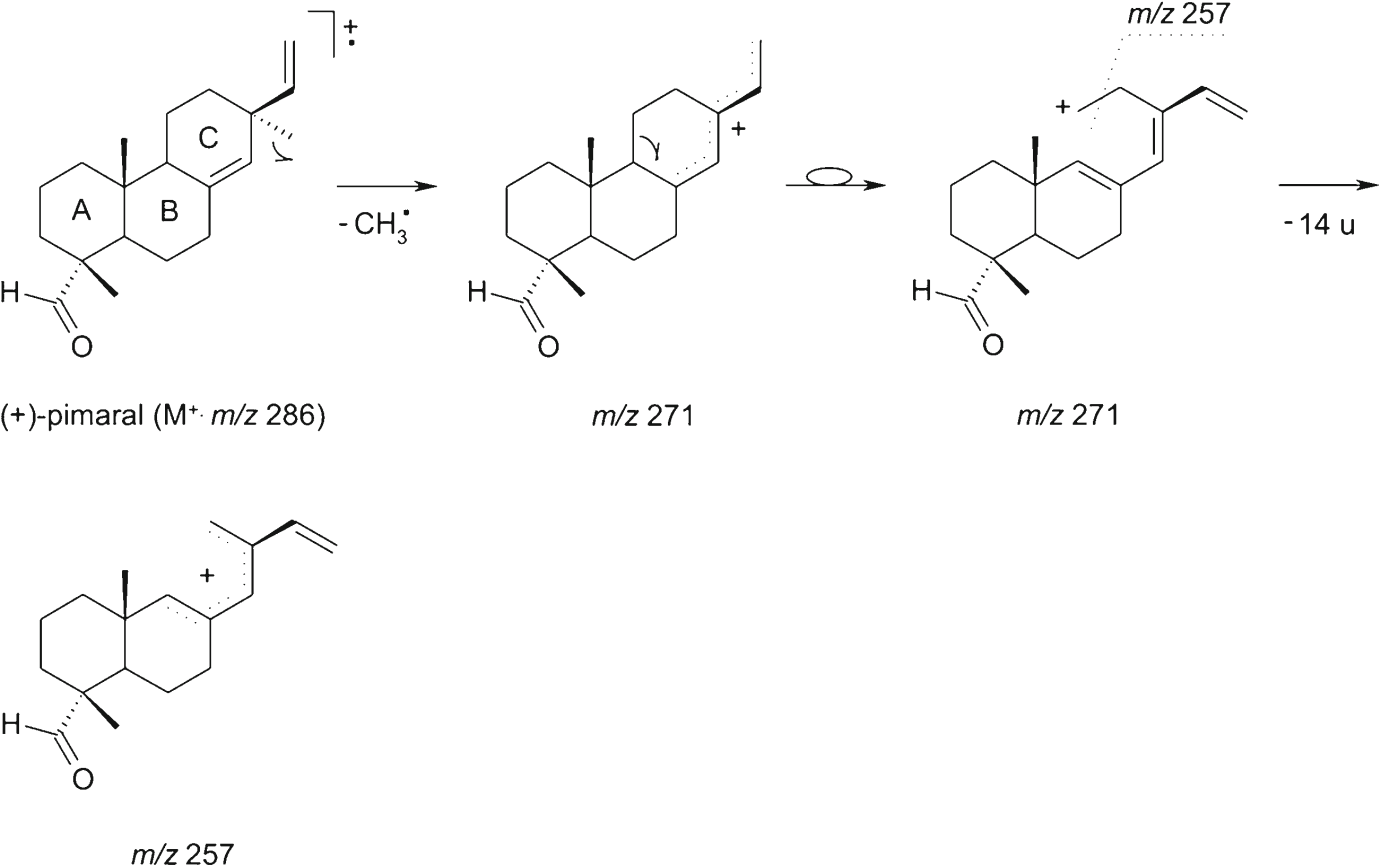
A plausible mechanism for the formation of relevant ions in the EI mass spectrum of (+)-pimaral 12

In the third retention time zone of TIC (Fig. 3)—covering a region between 22 and 34 min—a group of intense peaks are on display. Two peaks have been unambiguously assigned to manoyl oxide 11 and (+)-pimaral 12, based on the library matching and detailed interpretation of their mass spectra. These volatile constituents of the bark of *P. sylvestris*, chemically fall into the group of diterpenes. Diterpenes are ubiquitous in pine oleoresins—important forestry products traditionally obtained by tapping the bark of Pine trees and industrially processed into coatings, adhesives, fragrances and food gums (Breitmaier 2008; Rezzi et al. 2005). The fragmentation profiles of diterpenes were studied in depth elsewhere using metastable mass spectrometry (Enzel and Wahlberg 1969). However, in contrast to the old data suggesting that the key bond cleavage takes place in the A ring, the fragmentation reactions for diterpenes 11 and 12 more likely proceed via the cleavage of the C ring (Scheme 2). This would better rationalise the presence of the diagnostic ions in the first order mass spectra, e.g. even-electron ions at *m/z* 271, 257 for the volatile 12 (Fig. 5b). The total ion chromatogram (Fig. 3) shows additional peaks in the discussed retention time zone. These peaks, however, could not be identified unequivocally by the comparison with mass spectral libraries, since the matching indices where below 40%. In addition, the mass spectra of the unknown compounds from the volatile fraction were by far similar, and differed only in terms of the relative abundances. The identification of these unknowns deserves further scientific effort. While analysing the *P. sylvestris* bark sample with a SPME-GC/MS technique, we have noticed a number of weird peaks in the chromatographic trace (marked with asterisks in Fig. 3). Their presence has nothing to do with a sample, as they originated from the SPME polydimethylsiloxane cartridge bleeding. One of the contamination peaks which appeared at the retention time of 25.2 min was firmly assigned to diisooctyl phthalate, a characteristic mass spectrometry pollutant. Its usual sources are manifolds, but in our case the more likely source was a tubing system installed at the inlet and outlet of the sampling flow apparatus (Fig. 2).

Most of the 12 components we identified in the volatile emissions from the bark of *P. sylvestris* occurred also in other plant emissions and induced bioresponse in herbivore insect, including *M. galloprovincialis*. Terpenes α-pinene 1 and ∆-3-carene 2 were found virtually in all emissions, from needles (Heijari et al. 2011) to logs (Byers et al. 1985). The first one, α-pinene 1, was postulated to act as a host volatile used by some bark beetle species to locate suitable host trees (Pitman 1971). This kairomonal effect has been already utilised in the formulation of bait blends for insect traps (Gove et al. 2007). Specifically, it was a component of trap baits effective for *M. galloprovincialis* (Ibeas et al. 2007; Francardi et al. 2009). Both α-pinene 1 and ∆-3-carene 2 induced the electroantenographic (EAD) response in this insect (Weißbecker et al. 2006), although the latter was inactive towards other species (Weißbecker et al. 2004). Para-cymenene 3 was detected in the volatile fraction from whole pine trees (Weißbecker et al. 2006), twigs (Mumm et al. 2003) and needles (Mateus et al. 2010). It induced the EAD response in *M. galloprovincialis* (Weißbecker et al. 2006) and attracted an egg parasitoid of sawfly *Diprion pini* (Mumm et al. 2003).

Oxygenated terpenes α-terpineol 4 and verbenone 5 were also detected among volatiles from pine needles (Mateus et al. 2010) and from timber (Weißbecker et al. 2004; Fettkother et al. 2000). Both compounds have been proved to elicit a physiological response in spruce bark beetle *Dendroctonus micans* and trigger the aggregation of its larvae on the trees (Grégoire et al. 1991). They affected females of old home borer *Hylotrupes bajulus* in a wind tunnel bioassay (Fettkother et al. 2000), but induced no EAD response in this species (Weißbecker et al. 2004).

Among sesquiterpenes 6–10, α-longipinene 6 has not been detected while longifolene 7, γ-cadinene 9 and E-β-farnesene 8 were detected in bark and twig emissions of *P. sylvestris* (Mumm et al. 2003, 2005; Heijari et al. 2011). The latter compound occurred also in needle volatiles (Mateus et al. 2010). Pentadecane 10 was found only in the needle emissions (Mateus et al. 2010). The role of sesquiterpenes 6–10 in the plant–insect interaction is thought to be limited, because of their low vapour pressure and high reactivity towards atmospheric ozone. The atmospheric lifetime of these compounds is usually a few minutes (Rimetz-Planchon et al. 2011). However, longifolene 7 is as a notable exception since its atmospheric lifetime of several days (Atkinson and Arey 2003; Canosa-Mas et al. 1999) makes it a plausible signalling biomolecule. Indeed, recent biological studies revealed that a blend of longifolene **7** with α- and β-pinene released by larval *Monochamus alternatus* helps the *Pinus massoniana* parasitic nematode (*Bursaphelenchus xylophilus*) to build up a chemoecological relationship with its future vector insect (Zhao et al. 2007). The aliphatic sesquiterpenes E-β-farnesene 8 and pentadecane 10 serve as pheromones of various insects (Herrmann 2010). In the context of the insect–plant interactions, farnesene-type sesquiterpenes were demonstrated to be important insect juvenile hormones produced by some plants, e.g. *Pinus* species, to maintain their self-defence mechanisms (Sláma 1969). The sesquiterpnene γ-cadinene 9 was hypothesised to exhibit semiochemical activity (Mumm and Hilker 2005). In a laboratory bioassay, γ-cadinene 9 was the most attractive volatile to smaller European elm bark beetles (Millar et al. 1986).

Of the group of diterpenes identified in this work, manoyl oxide 11 has not been detected elsewhere, while (+)-pimaral 12 was found in essential oils obtained by steam distillation of freshly crushed trunks of *Pinus densiflora* and *Pinus tunbergii* (Sakai and Yamasaki 1990). These authors clearly demonstrated that vapours of (+)-pimaral 12 induced a physiological bioresponse in female cerambycid beetles *M. alternatus*.

It is clear that further bioassay research is necessary to evaluate the response of the black sawyer beetle *M. galloprovincialis*, and possibly other bark beetles, to the volatile components of emissions from the bark of *P. sylvestris*. We suggest special attention is paid to longifolene 7 and (+)-pimaral 12, which appear specific for bark emissions and were reported bioactive to insects.

## Conclusions

Solid-phase micro-extraction technique combined with gas chromatography/mass spectrometry was shown to be a powerful analytical method for the fast and reliable analysis of volatile organic compounds. In our work, SPME-GC/MS analysis was utilised with success to characterise the molecular composition of the volatile fraction emitted from the bark of *P. sylvestris*. A number of compounds were firmly identified based on the comparison with data from mass spectral libraries and, in most cases, by comparison with synthetic standards. A range of identified volatiles include: terpenes such as α-pinene, ∆-3-carene and para-cymenene; oxygenated terpenes such as α-terpineol and verbenone; sesquiterpenes such as α-longipinene, E-β-farnesene and γ-cadinene; as well as diterpenes such as manoyl oxide and (+)-pimaral. Of these, (+)-pimaral and longifolene are of particular interests owing to their plausible attracting capabilities towards black pine sawyer beetle *M. galloprovincialis*. Further research is required to evaluate this hypothesis and to select other molecular candidates from our work that are attractive for this insect.
